# AI awareness and the breakdown of daily recovery: a spillover pathway to work–family strain

**DOI:** 10.3389/fpubh.2025.1738073

**Published:** 2026-01-13

**Authors:** Xiaoyi Yi, Sameer Kumar

**Affiliations:** Asia Europe Institute, University of Malaya, Kuala Lumpur, Malaysia

**Keywords:** AI awareness, experienced sampling method, psychological detachment, trait resilience, work–family conflict

## Abstract

**Background:**

The growing adoption of AI in the workplace has emerged as a distinct occupational stressor, influencing employees’ psychological states and extending its effects into their family lives—a dimension rarely addressed by prior research.

**Objective and methods:**

Drawing on data collected from a final valid sample of 119 hotel frontline employees over 10 consecutive workdays via experience sampling (yielding 965 daily observations), this study investigates how AI awareness leads to work–family conflict (WFC). Specifically, we examine the mediating role of psychological detachment and the moderating function of trait resilience.

**Results:**

Our findings demonstrate that AI awareness increases WFC by impairing employees’ ability to detach from work. However, high levels of trait resilience reduce this negative pathway, supporting the buffering effect of personal resources.

**Conclusion:**

This study advances the literature on AI awareness by uncovering how it shapes employees’ work–family conflict and by positioning psychological detachment as a crucial recovery mechanism in this process.

## 1 Introduction

The hospitality industry is currently undergoing profound changes, characterized by the escalating integration of AI into its daily operational frameworks (1, 2). Hotels and service providers have increasingly adopted AI tools—including robotic assistants, intelligent automation, and customer interaction technologies (1)—to streamline operations and enhance service efficiency. AI and robotics are poised to transform the hospitality industry significantly, with predictions indicating that up to 95% of frontline roles could be automated. This shift is evidenced by the adoption of AI technologies in over 70% of hotels, aimed at enhancing efficiency in service delivery (3). However, this widespread adoption has also brought about new challenges, as this technological transformation introduces a new type of workplace pressure, which is referred to as AI awareness (4–6). AI awareness reflects employees’ recognition that the integration of artificial intelligence heightens internal competition, thereby triggering concerns about future employment stability and changes in professional responsibilities (1, 7).

Existing literature indicates that employees’ awareness of AI might adversely affect multiple job-related outcomes, such as proactive service behavior (8), employee silence (9), performance adaptivity (10), voice behavior (11, 12), and work withdraw behavior (2). Although research into the adverse effects of AI awareness is nascent, existing studies predominantly examine work-related outcomes, often neglecting how it may affect the family lives of employees (13). Given that work–family balance is a critical factor influencing employees’ well-being, job satisfaction, and overall performance, understanding its antecedents—particularly the relationship between AI awareness and work–family conflict—is essential for fostering employee resilience and ensuring organizational effectiveness in the evolving technological landscape (14). According to the spillover model, negative work experiences, such as anxiety due to AI awareness, can extend into one’s family life, potentially resulting in work–family conflict (13). It is important to note that while the work–family interface is multidimensional—comprising both positive and negative spillover in both directions (15)—this study specifically investigates negative work-to-family spillover. We conceptualize this dimension as the extent to which work-related stressors impede functioning in the family domain. Yet, limited knowledge exists regarding how AI awareness affects frontline employees’ home lives—especially in terms of the underlying mechanisms tied to work–family conflict and the influence of possible moderators. This study investigates how AI awareness affects employees’ experience of work–family conflict by focusing on psychological detachment as a key explanatory mechanism. Essential for recovery, psychological detachment is the process of withdrawing mentally from work-related concerns once the workday ends. This process entails a mental disconnection from job responsibilities while away from the workplace (16, 17). However, despite growing interest in the impact of AI-related stressors, prior research has yet to integrate this recovery-based mechanism into the study of AI awareness. This omission is noteworthy because AI awareness, as a future-oriented and cognitively intrusive stressor, tends to persist beyond work hours and continuously occupies mental bandwidth (6). Unlike acute stressors that dissipate after the workday, AI awareness involves anticipatory anxiety about job displacement and long-term career insecurity, which can spill over into personal life.

Furthermore, the stressor-detachment perspective highlights that individuals’ internal strengths can buffer the negative influence of occupational stress on recovery (18, 19). Among these strengths, trait resilience plays a critical role in helping employees mentally disconnect from work demands when under pressure (8, 17). Research has shown that being aware of AI-related risks may trigger harmful emotions in frontline workers. These emotions often bleed into nonwork domains and intensify conflicts between work and family roles (6). The capacity to recover from strain and sustain emotional regulation—core features of trait resilience (20)—is crucial for alleviating psychological burdens introduced by AI-related pressures in organizational settings.

**Figure 1:**
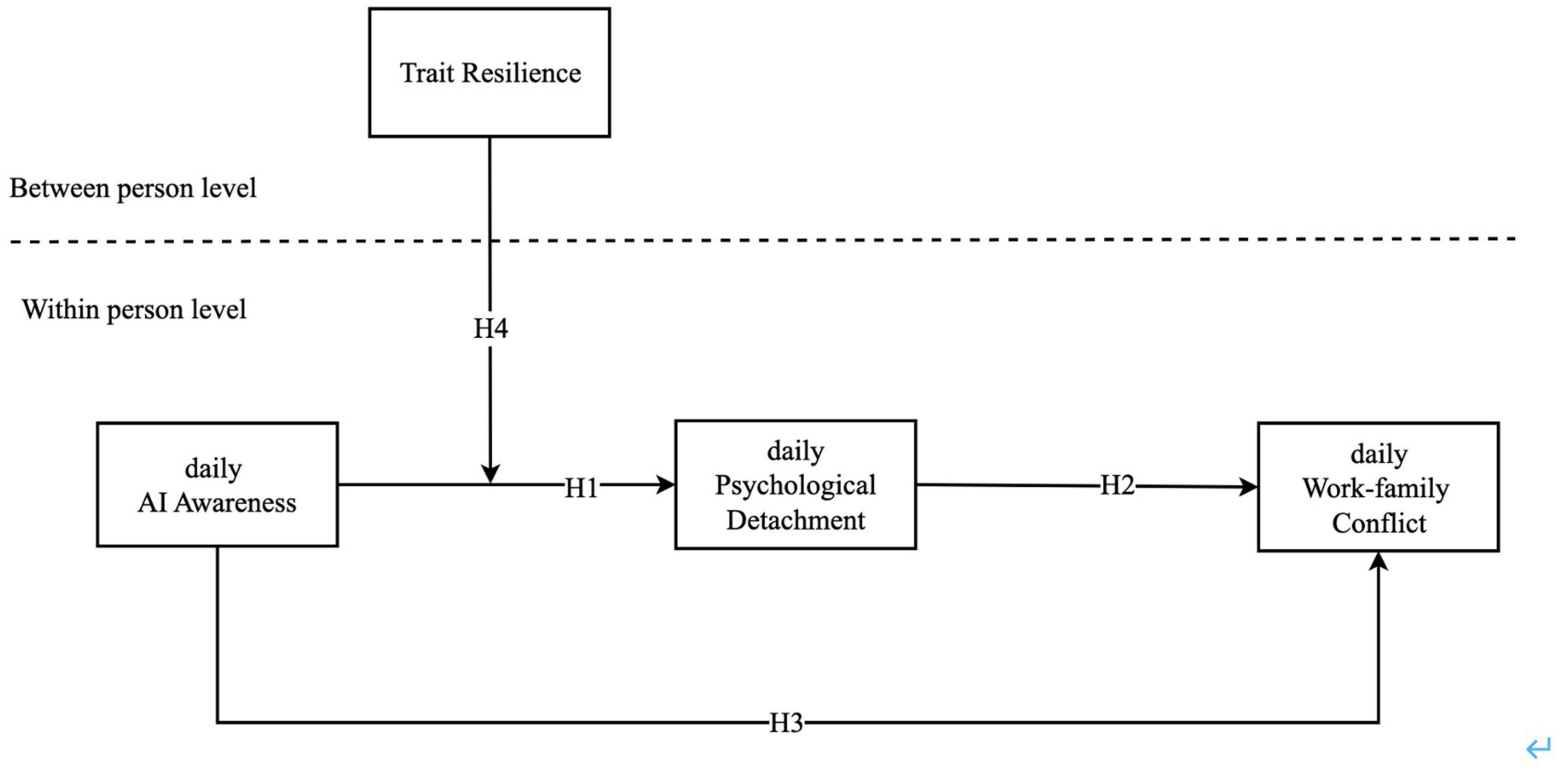
Conceptual framework.

Informed by prior theoretical and applied considerations, this work advances understanding of AI awareness in three principal ways. Foremost, it broadens the focus from workplace implications to include the family domain, providing a more integrated view of how AI-related stress permeates both occupational and personal spheres. Second, our research clarifies that psychological detachment acts as a mediating mechanism, illustrating how AI awareness exacerbates WFC by disrupting recovery processes, ultimately affecting employees’ off-work well-being. Third, our study advances knowledge of the boundary conditions influencing AI awareness by exploring how trait resilience moderates its negative impacts on psychological detachment and WFC. Figure 1 illustrates the model hypothesized in our study.

## 2 Theoretical framework and hypotheses

### 2.1 The stressor–detachment model

This research adopts the stressor–detachment framework (19) to explain how AI awareness influences the balance between work and family life among frontline hotel staff. Within this perspective, AI awareness is conceptualized as a workplace stressor that hampers employees’ ability to mentally disengage from job demands after working hours, thereby heightening strain that extends to both professional and personal domains (21). Psychological detachment functions as a vital recovery process, enabling individuals to restore depleted resources and offset the adverse consequences of occupational stressors (16, 17). Investigating this mechanism’s role within our model provides deeper insights into how AI awareness affects employee well-being, particularly under the high-stress conditions prevalent in the hospitality industry.

Among frontline hotel workers, perceiving AI as a workplace stressor can trigger pronounced job insecurity and uncertainty (4, 22). Prolonged exposure to such stress undermines the ability to mentally detach from work during leisure time, creating conditions for elevated stress. Persistent engagement with work concerns outside office hours not only intensifies negative emotional states but also heightens the probability of conflict between work and family roles, as evidenced by prior research (21). Given that frontline hotel employees must consistently engage with customers and handle the additional pressures associated with AI integration, their need for effective psychological detachment becomes even more critical for maintaining overall well-being and preserving harmony in their family lives.

### 2.2 Al awareness and psychological detachment

Psychological detachment is characterized as “the individual’s perception of being removed from the work environment” (23). This involves abstaining from work-related tasks, like reviewing emails, and steering clear of work-centric thinking during downtime (19). As a key psychological mechanism, detachment helps elucidate the pathway through which job stressors give rise to detrimental effects (19, 24). In this study, AI awareness is conceptualized as a significant job stressor, particularly for frontline hotel employees who face ongoing pressure from AI integration and automation (4). Drawing on the stressor–detachment model, heightened awareness of AI technologies may amplify anxiety and perceptions of job instability, thereby restricting workers’ mental separation from job demands during their personal time. In the hospitality sector, heightened awareness of AI triggers persistent ruminative thoughts and concerns among employees, compromising their ability to mentally disengage from their work responsibilities (2). This impaired detachment often leads to adverse effects, as stress and anxieties about AI integration prevent employees from fully disengaging during off-hours (6).

Grounded in the Conservation of Resources perspective, this relationship suggests that employees, faced with work-induced stressors, must allocate their finite personal resources to manage escalating job demands, potentially leading to resource depletion (25). When facing the perceived threat of AI replacing their roles, hotel employees often expend significant physical and mental energy to manage these new challenges, including the need to continuously improve skills and adapt to AI advancements (2). This continuous strain can challenge employees’ capacity to disengage mentally from their professional duties, as they remain preoccupied with worries about AI integration (5). The ongoing resource depletion caused by this preoccupation inhibits effective psychological detachment, preventing employees from adequately recovering from work stress (17). Thus, AI awareness is proposed to negatively impact psychological detachment, leading to persistent strain for these employees.

*H1*: AI awareness negatively impacts psychological detachment.

### 2.3 Psychological detachment and WFC

For frontline hotel employees, whose roles are characterized by intense demands and continuous customer interaction, the experience of work–family conflict (WFC) is a significant occupational challenge. WFC is understood as a fundamental mismatch between the obligations of one’s profession and the capacity to fulfill personal family duties (26). To mitigate this issue, the ability to psychologically detach from work-related thoughts and tasks during non-work time becomes a critical recovery mechanism. This conflict’s impact on work-life harmony is not uniform; it can manifest through time-based, strain-based, and behavior-based dimensions, each creating distinct pressures on an individual’s personal sphere (27).

Time-based work–family conflict refers to the situation in which employees’ cognitive focus on job-related issues persists beyond official working hours, encroaching on the time that could otherwise be allocated to family roles (28). Within the hospitality industry, this form of conflict is particularly prevalent among frontline personnel. Frontline employees often find themselves thinking about unresolved customer issues or trying to learn and adapt to AI tools during their supposed downtime. For example, an employee may spend their evenings acquiring AI-related knowledge to enhance job performance or integrating new AI tools into their workflow, consequently leaving limited time for fulfilling family responsibilities (2, 12). This ongoing preoccupation leads to time-based WFC, where the time needed for family engagement is compromised. Second, strain-based WFC occurs when the emotional and physical stress accumulated from work prevents employees from effectively participating in family activities (27). Frontline hotel employees, who face demanding customers and uncertainties related to AI integration, often worry about job security (5). Given that their job is a crucial personal resource, concerns about AI replacing their roles can exacerbate their stress (29). Additionally, negative emotions experienced during the workday, fueled by AI awareness, can extend into the home, further intensifying this strain (6, 26). As a result, employees may be less emotionally available for their families, making it challenging to fulfill their roles at home, thereby contributing to strain-based WFC. Lastly, behavior-based WFC occurs when employees face difficulty shifting from their work role to their family role, resulting in role incompatibility (27). Frontline hotel employees, who must be attentive, efficient, and responsive at work, may find that an inability to mentally detach from these professional behaviors leads to unsuitable interactions at home (30). In summary, frontline hotel employees often experience increased WFC due to impaired psychological detachment.

*H2*: Psychological detachment is negatively associated with work–family conflict.

### 2.4 The mediating role of psychological detachment

Extant literature reveals that stress originating from the workplace can impair employees’ family-related quality of life by constraining their capacity to recuperate. While the stressor-detachment model does not directly address technological stressors, it offers a lens through which we can explore their potential impact on both work and family life. Within this framework, elevated AI awareness—often marked by concerns over job security and the threat of AI replacement (4, 7, 22)—acts as a persistent workplace stressor for frontline hotel employees. This intensified awareness can evoke detrimental emotional reactions that interfere with employees’ ability to cognitively disengage from work-related concerns, even beyond their scheduled shifts (6). Low psychological detachment manifests in behaviors like ruminating on work issues, using personal time to learn about AI, or planning AI integration into work tasks (2, 6, 29). Such activities consume time that might otherwise be allocated to family duties, thus exacerbating conflicts related to time management in work–family balance. Moreover, negative emotions and stress stemming from AI awareness can spill over into family life, exacerbating strain-based WFC. The inability to fully step away from work-related habits can also lead to behavior-based WFC, where work behaviors and attitudes clash with the expectations of the family role. In sum, AI awareness is likely to positively predict WFC through decreased psychological detachment.

*H3*: AI awareness will indirectly affect WFC through psychological detachment.

### 2.5 The moderating role of trait resilience

The stressor–detachment framework posits that the extent to which psychological detachment alleviates workplace stressors is contingent upon employees’ personal resources (18, 19). In light of this, scholars have increasingly advocated examining how individual differences interact with AI awareness as situational contingencies (2, 6, 9–11). Trait resilience serves as a vital personal asset that enhances employees’ ability to navigate stress and overcome adversity (31). Research indicates that individuals with higher trait resilience are more proficient in handling adverse work experiences (17, 32). Previous research has demonstrated that resilience helps employees cope with various work-related stressors (17, 33); however, its role in mitigating the specific challenges posed by AI awareness remains unclear.

Empirical evidence suggests that employees with stronger trait resilience possess greater capacity to navigate adverse workplace situations (32). Resilience has been shown to buffer the impact of diverse work-related stressors, enabling employees to adapt and maintain functioning under challenging conditions (17, 33). Our study investigates how trait resilience interacts with AI awareness, shedding light on its effectiveness in buffering AI-induced stress. According to previous research, trait resilience helps individuals cope not only with major life challenges but also with daily, fluctuating stressors (17, 20, 31). In the context of AI awareness, employees with greater trait resilience are better able to disengage from work, reducing its detrimental effects on their psychological well-being. This is because resilient employees tend to maintain a calm and positive outlook in stressful situations (17), allowing them to prevent negative experiences related to AI awareness from lingering and affecting their recovery. Conversely, those with lower trait resilience may find it difficult to disengage from stressful situations, prolonging their emotional response and impeding recovery.

*H4*: Trait resilience is proposed to buffer the detrimental association between AI awareness and psychological detachment, such that individuals with higher resilience are less affected by AI awareness in terms of their capacity to mentally disengage from work.

## 3 Methodology

### 3.1 Participants and procedure

To investigate the effects of AI awareness on frontline hospitality employees, this study was conducted within five luxury hotels along China’s eastern coast. The selection of these specific establishments was predicated on their significant investment in and operational integration of advanced AI technologies, such as service robots and automated concierge systems. This criterion provided a suitable real-world setting for our research. The data collection was strategically timed to coincide with the peak tourist season, from June to August 2024, to facilitate the observation of human-AI interactions during a period of high operational intensity and frequent customer engagement. Ethical and procedural integrity was maintained through close collaboration with the human resources department of each hotel. Participants were onboarded via a secure mobile platform, adhering to stringent data protection standards. Prior to commencement, all individuals received a comprehensive briefing on the research objectives, were guaranteed absolute confidentiality of their responses, and were explicitly informed of their right to withdraw from the study at any point without penalty.

Our data collection protocol employed a two-stage, experience sampling methodology (ESM). The first stage involved a foundational survey designed to capture stable, between-person variables, including trait resilience and key demographic information (e.g., gender, age, marital status, job tenure). This survey was distributed via links on the WeChat platform, and a remuneration of 30 Chinese Yuan (approximately $4.39 USD) was provided as compensation for its completion. Two weeks following the initial survey, the second stage commenced. This phase required participants to engage in a daily diary study for 10 consecutive workdays. Each day consisted of two distinct survey instances. The first, administered toward the end of their work shift, assessed transient, state-level variables such as daily AI awareness (AIA), job demand (JD), and negative emotions (NE). The second instance, completed before bedtime, measured psychological detachment (PD) and work–family conflict (WFC). To ensure high participant retention and data quality throughout this intensive 10-day period, a further incentive of 70 Chinese Yuan (approximately $10.23 USD) was awarded upon the successful completion of all daily surveys.

From an initial pool of 131 frontline hotel employees who consented to participate, 12 were subsequently excluded from the final analysis. The basis for this exclusion was the failure to meet the minimum data requirement of completing surveys for three consecutive days, a threshold deemed necessary for reliable within-person variance analysis (34). This refinement process yielded a final valid sample of 119 participants, from whom a total of 965 daily survey responses were collected over the course of the study. An analysis of the final sample reveals a specific demographic composition. A majority of participants were female (57.4%) and married (66.3%). Educational attainment was led by individuals with college degrees (54.5%), with a significant portion also having completed high school (34.7%). The age distribution was concentrated in the 25–34 year bracket, which included 64.4% of respondents. Furthermore, the most represented tenure group consisted of employees with 1 to 2 years of experience (41.6%), and a substantial 69.3% reported a monthly income between 3,000 and 7,000 RMB.

### 3.2 Measures

Each variable in this study was measured using a 5-point Likert scale, with responses ranging from 1 (“strongly disagree”) to 5 (“strongly agree”). AI Awareness (AIA) was assessed using four items modified from the scale developed by Brougham and Haar (7), such as “Today, I am personally worried that what I do now in my job will be able to be replaced by AI.” Psychological Detachment (PD) was evaluated using four items derived from the scale by Sonnentag and Fritz (24), including “Today, when I came home today, I forgot about work.” Work–Family Conflict (WFC) was measured using four items adapted from Grzywacz and Marks (15), such as “Today, my job reduces the effort I can give to activities at home.” Trait resilience (TR) was evaluated using six items adapted from Smith et al. (35), including “I tend to bounce back quickly after hard times.” A 5-point Likert scale (1 = “strongly disagree,” 5 = “strongly agree”) was applied to all constructs. AI Awareness (AIA) was assessed through four adapted items from Brougham and Haar (7), such as “Today, I worry that AI could take over my current work.” Psychological Detachment (PD) was measured with four items from Sonnentag and Fritz (24), for example, “Today, after arriving home, I disengaged completely from work.” Work–family Conflict (WFC) was evaluated via four items from Grzywacz and Marks (15), including “Today, my job reduced the effort I could invest at home.” Trait resilience (TR) was gauged using six items from Smith et al. (35), such as “I recover rapidly after setbacks.” Control variables included gender, age, job tenure, marital status, education level, job demand (JD), and negative emotions (NE), as these variables could potentially influence the relationships under investigation. JD was measured with five items from Spector and Jex (36), for example, “Today, my job involved a variety of complex tasks.” NE was measured using three items, such as “Today, I felt anxiety,” adapted from Bono et al. (37).

### 3.3 Analyses

In analyzing the complex dynamics of our dataset, characterized by daily observations nested within individual participants, we employed a two-level multilevel modeling approach. To execute our two-level MLM, we utilized the “lme4” package within the R statistical environment, leveraging its robust capabilities for managing such complex data structures. At the within-person level (Level 1), we examined the daily variables including AI awareness (AIA), psychological detachment (PD), and work–family conflict (WFC), as well as job demand (JD) and negative emotions (NE). Each participant’s daily scores were centered around their mean to remove any between-person effects and isolate the day-to-day variability. At the between-person level (Level 2), predictors were grand-mean centered to assess how individual differences in trait resilience influenced the hypothesized relationships, providing insight into how personal resources shape employees’ responses to AI awareness within the organizational context.

To assess the reliability of our daily variables, we calculated within-person reliability (Rc) for each construct to ensure consistent measurement throughout the study duration. AI awareness (AIA) showed a high reliability of 0.775, indicating that employees consistently reported their perception of AI-related stress across days. Psychological detachment (PD) recorded a reliability of 0.772. Work–family conflict (WFC) showed an Rc of 0.762, suggesting that the experiences of work interfering with family life were consistently reported across the study period. Negative emotions (NE) and job demand (JD) both demonstrated adequate reliability with Rc values of 0.716 and 0.714, respectively. All Rc values exceeded the widely recognized benchmark of 0.70, affirming the robustness of our measurements and confirming the reliability of our daily data collection.

### 3.4 Between and within person variances

An overview of variable distributions and interrelations is provided in Table 1, which summarizes descriptive statistics and correlation patterns. During the preliminary stage, null models were constructed individually for each variable, enabling the separation of variance into components attributable to within-person fluctuations and between-person differences (see Appendix Table A1 for detailed variance components). This step is crucial in multilevel modeling to determine the appropriateness of using hierarchical techniques for subsequent analyses. The ICC was calculated for the daily variables of AIA, PD, WFC, negative emotions, and job demand to quantify the extent of between-person variance. Analysis of the unconditional model showed that part of the variance in all key measures originated from daily fluctuations within individuals. For AIA, PD, WFC, NE, and JD, the ICCs were 0.295, 0.291, 0.279, 0.234, and 0.232, implying that 23.2–29.5% of total variance reflected stable differences across participants. The remainder was due to day-to-day variation or measurement imprecision, providing a strong rationale for employing multilevel techniques to test the study’s hypotheses.

**Table 1:** Descriptive statistics and correlation matrix.

Variable	M	SD	1	2	3	4	5	6	7	8	9	10
Level 2: Between person level												
1. Sex	1.55	0.50	—									
2. Age	1.94	0.59	0.04	—								
3. Tenure	2.08	0.90	0.01	−0.07^*^	—							
4. Edu	2.51	0.76	0.05	−0.16^***^	−0.09^**^	—						
5. MT	0.66	0.48	−0.13^***^	−0.02	−0.11^**^	−0.05	—					
6. TR	2.93	1.08	−0.11^**^	0.07^*^	0.11^**^	−0.09^**^	0.05	—				
Level 1: Within person level												
7. JD	3.03	0.99	0.08^*^	−0.04	0.01	−0.11^**^	−0.04	0.00	—			
8. NE	3.47	1.01	−0.03	−0.08^*^	−0.07^*^	−0.03	0.11^***^	0.17^***^	−0.09^**^	—		
9. AIA	3.24	1.07	0.01	0.19^***^	−0.05	−0.17^***^	0.03	0.02	0.14^***^	−0.05	—	
10. PD	2.96	0.81	0.00	−0.07	−0.07^*^	−0.06	0.03	0.01	0.05	0.02	−0.15^***^	—
11. WFC	2.96	1.13	−0.00	0.08^*^	0.03	−0.14^***^	−0.03	0.06	0.01	0.03	−0.22^***^	0.35^***^

* *p* < 0.05. ** *p* < 0.01. *** *p* < 0.001. AIA, AI awareness; PD, psychological detachment; WFC, work–family conflict; TR, trait resilience; JD, job demand; and NE, negative emotions.

### 3.5 Multilevel confirmatory factor analysis

Construct distinctiveness was examined via multilevel confirmatory factor analysis in R (“lavaan” package; Table 2). The six-factor specification—AIA, PD, WFC, TR, JD, and NE—showed robust fit (χ^2^/df = 4.927, CFI = 0.941, TLI = 0.933, RMSEA = 0.067, SRMR = 0.035), evidencing clear differentiation among variables. Alternative structures performed notably worse; for example, merging AIA, PD, and WFC into a five-factor model (*χ*^2^/df = 20.651, CFI = 0.696, TLI = 0.663, NFII = 0.686, IFI = 0.697, GFI = 0.623, RMSEA = 0.149, SRMR = 0.118) or condensing all variables into a one-factor model (*χ*^2^/df = 52.76, CFI = 0.183, TLI = 0.112, NFI = 0.182, IFI = 0.184, GFI = 0.374, RMSEA = 0.242, SRMR = 0.235) yielded substantially inferior fit statistics, reinforcing the six-factor model’s validity.

**Table 2:** Multilevel confirmatory factor analysis.

Model fit	*χ*^2^/df	CFI	TLI	NFI	IFI	GFI	AGFI	RMSEA	SRMR
Six-factor	4.927	0.941	0.933	0.927	0.941	0.885	0.858	0.067	0.035
Five-factor 1	15.947	0.772	0.744	0.761	0.772	0.695	0.63	0.13	0.114
Five-factor 2	13.072	0.816	0.793	0.804	0.816	0.723	0.664	0.117	0.089
Four-factor 1	28.436	0.576	0.529	0.568	0.576	0.51	0.413	0.176	0.179
Four-factor 2	20.651	0.696	0.663	0.686	0.697	0.623	0.548	0.149	0.118
Three-factor 1	35.76	0.457	0.404	0.451	0.458	0.458	0.358	0.199	0.194
Three-factor 2	32.086	0.514	0.467	0.507	0.515	0.49	0.396	0.188	0.189
Two-factor	44.375	0.318	0.256	0.314	0.319	0.402	0.295	0.222	0.222
One-factor	52.76	0.183	0.112	0.182	0.184	0.374	0.265	0.242	0.235

Six-factor: AIA, PD, WFC, TR, JD, NE; five-factor 1: AIA + PD, WFC, TR, JD, NE; five-factor 2: AIA, PD + WFC, TR, JD, NE; four-factor 1: AIA, PD + WFC + TR, JD, NE; four-factor 2: AIA + PD + WFC, TR, JD, NE; three-factor 1: AIA + PD + WFC + TR, JD, NE; three-factor 2: AIA, PD, WFC + TR + JD + NE; two-factor: AIA + PD + WFC, TR + JD, NE; one-factor: AIA + PD + WFC + TR + JD + NE; AIA, AI awareness; PD, psychological detachment; WFC, work–family conflict; TR, trait resilience; JD, job demand; and NE, negative emotions.

## 4 Results

### 4.1 Reliability and convergent validity

The study evaluated the reliability and convergent validity of each construct (Table 3). Reliability was confirmed as all alpha coefficients were above 0.70. CR values also satisfied the 0.70 threshold suggested by Fornell and Larcker (38), and AVE scores exceeded the 0.50 criterion, indicating convergence among indicators. Together, these metrics demonstrate that the measurement framework reliably and validly reflects the constructs under investigation, supporting its application in further hypothesis testing.

**Table 3:** Descriptive statistics.

Reliability and convergent validity	Loading	S.E.	*z*	Cronbach’s alpha	CR	AVE
Within-person level						
AIA≃AIA1	0.755	0.035	25.44	0.886	0.888	0.668
AIA≃AIA2	0.729	0.037	24.232			
AIA≃AIA3	0.894	0.035	32.666			
AIA≃AIA4	0.863	0.035	30.916			
PD≃PD1	0.941	0.035	36.91	0.932	0.939	0.798
PD≃PD2	0.921	0.035	35.593			
PD≃PD3	0.914	0.037	35.122			
PD≃PD4	0.746	0.035	25.635			
WFC≃WFC1	0.921	0.033	35.185	0.923	0.922	0.747
WFC≃WFC2	0.898	0.031	33.731			
WFC≃WFC3	0.805	0.036	28.416			
WFC≃WFC4	0.837	0.037	30.116			
JD≃JD1	0.92	0.035	34.815	0.894	0.898	0.644
JD≃JD2	0.858	0.03	31.099			
JD≃JD3	0.824	0.032	29.216			
JD≃JD4	0.693	0.034	22.873			
JD≃JD5	0.66	0.036	21.438			
NE≃NE1	0.857	0.027	31.565	0.842	0.843	0.642
NE≃NE2	0.972	0.027	38.992			
NE≃NE3	0.928	0.029	35.902			
Between-person level						
TR≃TR1	0.838	0.029	30.387	0.943	0.944	0.738
TR≃TR2	0.836	0.034	30.248			
TR≃TR3	0.864	0.035	31.911			
TR≃TR4	0.856	0.035	31.384			
TR≃TR5	0.893	0.033	33.703			
TR≃TR6	0.859	0.032	31.563			

AIA, AI awareness; PD, psychological detachment; WFC, work–family conflict; TR, trait resilience; JD, job demand; and NE, negative emotions.

### 4.2 Hypothesis test

As summarized in Table 4, the multilevel analysis provided consistent support for all three hypotheses. Model 2 showed that AIA was significantly and negatively related to PD (*γ* = −0.192, *p* < 0.001), consistent with Hypothesis 1. Model 7 indicated that PD had a significant negative association with WFC (*γ* = −0.344, *p* < 0.001), confirming Hypothesis 2. For Hypothesis 3, mediation analysis revealed that AIA was positively associated with WFC in Model 6 (*γ* = 0.293, *p* < 0.001). When PD was added in subsequent models, it emerged as a significant negative predictor of WFC, while the effect of AIA was reduced yet remained significant (Model 8: AIA *γ* = 0.233, *p* < 0.001; PD *γ* = −0.309, *p* < 0.001), indicating partial mediation. A bootstrap test with 5,000 iterations confirmed the indirect pathway from AIA to WFC through PD (effect = 0.059, SE = 0.013, z = 4.557, *p* < 0.001, 95% CI [0.035, 0.085]).

**Table 4:** Regression models.

Variable	Psychological detachment				Work–family conflict			
	M1	M2	M3	M4	M5	M6	M7	M8
(Intercept)	3.338^***^	3.643^***^	3.593^***^	4.290^***^	3.246^***^	4.096^***^	1.625^**^	2.495^***^
Control variables								
Sex	0.032	0.036	0.042	0.039	0.015	0.027	−0.000	0.011
Age	−0.101	−0.070	−0.074	−0.078	0.100	0.188	0.150	0.218^*^
Edu	−0.062	−0.079	−0.077	−0.075	−0.196^*^	−0.242^**^	−0.165^*^	−0.208^**^
Tenure	−0.076	−0.083	−0.086	−0.096	0.022	0.001	0.059	0.038
MT	0.025	0.031	0.030	0.041	−0.080	−0.063	−0.092	−0.077
JD	0.013	0.025	0.025	0.031	−0.043	−0.011	−0.049	−0.022
NE	0.009	0.005	0.001	−0.000	0.037	0.026	0.032	0.023
Independent variable								
AIA		−0.192^***^	−0.192^***^	−0.201^***^		0.293^***^		0.233^***^
Moderator								
TR			0.047	0.047				
Interaction								
AIA × TR				0.133^***^				
Mediator								
PD							−0.344^***^	−0.309^***^
Marginal *R*^2^	0.014	0.032	0.033	0.041	0.025	0.092	0.141	0.189
Conditional *R*^2^	0.306	0.309	0.312	0.317	0.299	0.356	0.377	0.424
Random effects								
*τ*_00_	0.194	0.183	0.185	0.183	0.358	0.345	0.307	0.309
*σ* ^2^	0.462	0.455	0.455	0.451	0.917	0.845	0.810	0.758

* *p* < 0.05. ** *p* < 0.01. *** *p* < 0.001; AIA, AI awareness; PD, psychological detachment; WFC, work–family conflict; TR, trait resilience; JD, job demand; and NE, negative emotions.

**Figure 2:**
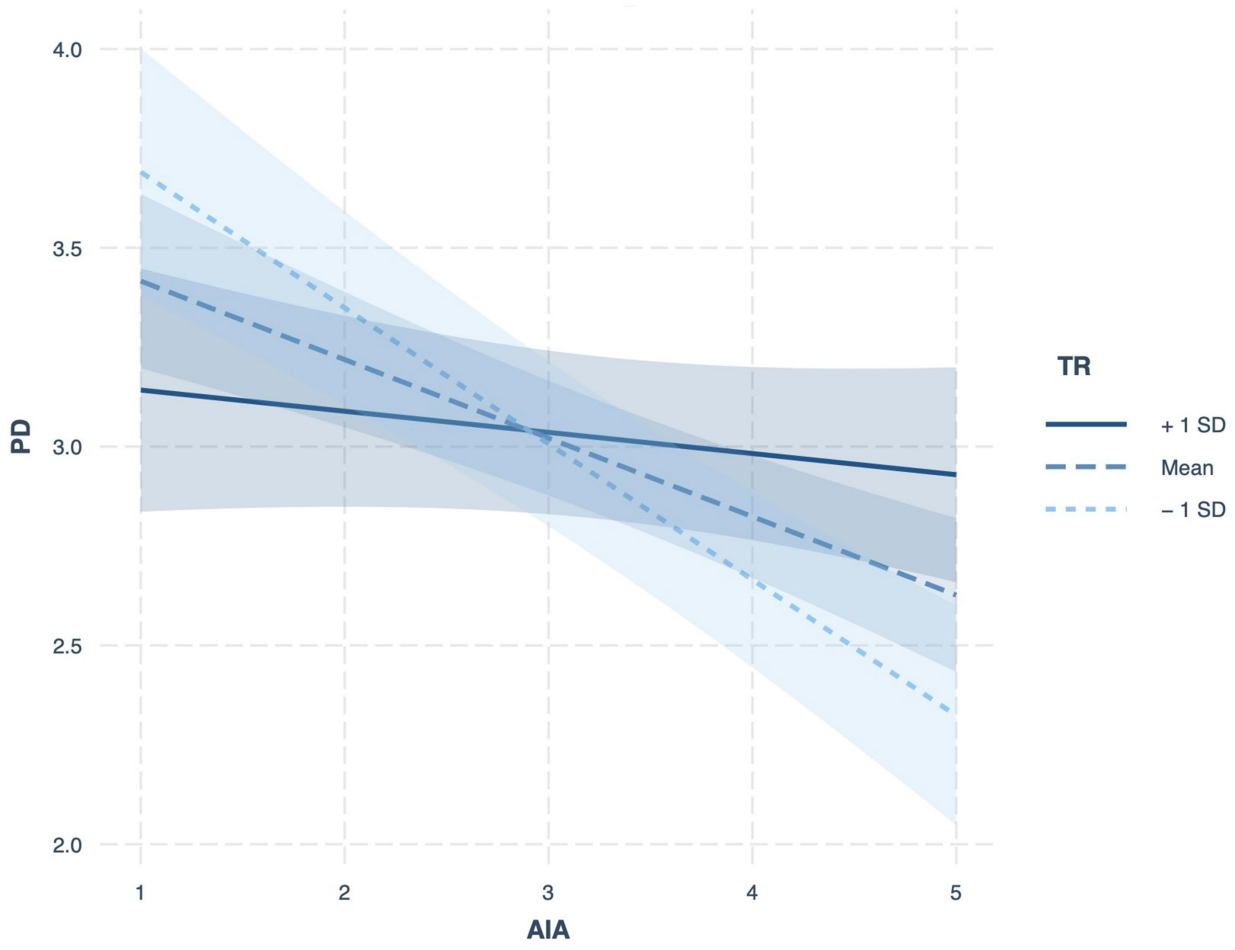
Moderating effect of TR.

**Figure 3:**
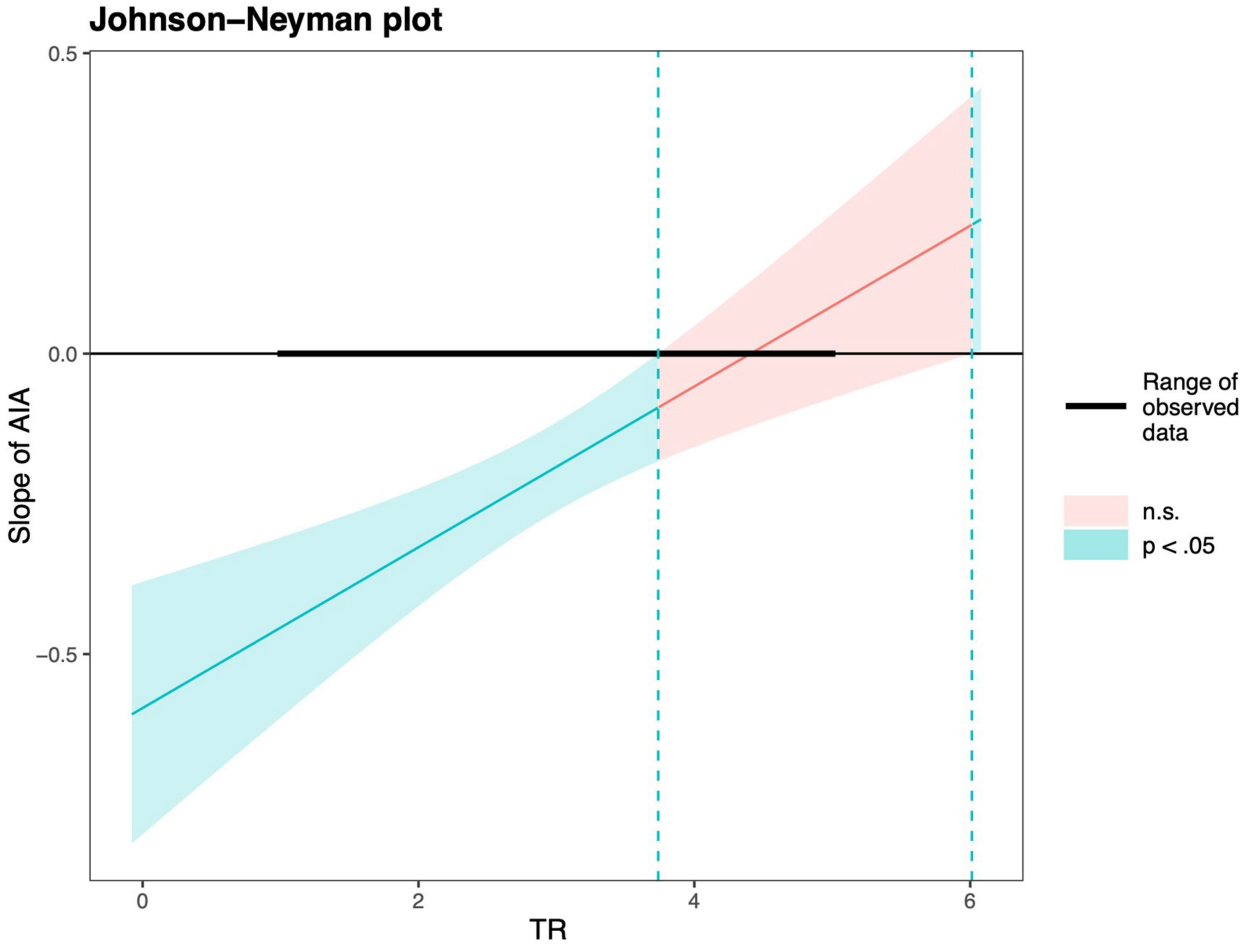
Johnson-Neyman analysis.

Hypothesis 4 anticipated that TR would serve as a boundary condition in the AIA–PD relationship, attenuating the detrimental effect of AIA when resilience is higher. Evidence from Model 4 showed a significant positive interaction term (*γ* = 0.133, *p* < 0.001). Probing this interaction via simple slope analysis (Figure 2) revealed that the negative association between AIA and PD was strongest at low TR (*γ* = −0.342, *p* < 0.001), weaker at average TR (*γ* = −0.197, *p* < 0.001), and non-significant at high TR (*γ* = −0.053, *p* = 0.30). These patterns align with Hypothesis 4, with Johnson–Neyman results clarifying that the buffering effect of TR renders the AIA–PD link insignificant above a certain resilience threshold (Figure 3). Specifically, when TR values were outside the interval [3.74, 6.01], the effect of AIA on PD was statistically significant (*p* < 0.05). However, within this range, the effect was not significant.

## 5 Conclusion

The present study uncovers that the effect of AI awareness on work–family conflict is transmitted through employees’ psychological detachment from work. Moreover, resilience plays a critical buffering role: employees exhibiting higher resilience demonstrate greater capacity to withstand the stress associated with AI awareness, allowing them to sustain detachment from work-related demands.

### 5.1 Theoretical implication

Firstly, our research enhances the AI awareness literature by extending its focus to encompass effects in the family context, thereby providing a more comprehensive view of its broader implications beyond the workplace. Prior research has largely explored the implications of AI awareness with a focus on work-related outcomes, such as proactive service behavior (8), employee silence (9), and voice behavior (11, 12), often overlooking the complex spillover effects it can have on employees’ personal lives. Our study expands on these findings by demonstrating that AI awareness, as a persistent workplace stressor, hinders frontline hotel employees’ ability to mentally detach from work, thereby exacerbating work–family conflict.

The second contribution of this study lies in uncovering a new pathway by which AI awareness impacts the work–family interface. It is critical to acknowledge that the work–family interface is multidimensional. As conceptualized by (15), spillover is not a singular construct but comprises four distinct dimensions: positive and negative spillover from work to family, and positive and negative spillover from family to work. While we recognize this complexity, our study specifically isolates the negative work-to-family spillover dimension. This focus is theoretically driven by the stressor-detachment model, which posits that workplace stressors deplete the cognitive and emotional resources necessary for functioning at home (19). By narrowing our lens to this specific dimension, we provide a more precise understanding of how AI awareness operates as a workplace ‘barrier’—a concept aligned with ecological perspectives that link environmental demands to negative spillover. We identify psychological detachment as the critical mediating mechanism in this specific pathway. Unlike previous studies that may have conflated general work–family conflict with emotional transmission (6), the present study shifts the focus beyond purely emotional pathways to consider a key r recovery process—psychological detachment—as the mechanism through which AI awareness exacerbates work-to-family conflict.

Third, this research extends the emerging scholarship on AI awareness by elucidating the conditions under which it intensifies or alleviates its impact on frontline employees. The impact of workplace stressors, such as AI awareness, varies among employees (5, 10). Given the accelerated incorporation of AI technologies in the hospitality sector and their considerable influence on employee experiences, existing literature increasingly emphasizes the necessity of probing the underlying factors that govern AI awareness (10, 11). Previous research has mainly focused on leadership factors and organizational-level variables as boundary conditions, such as organizational support (12), and empowering leadership (6). Research has rarely explored the ways in which personality characteristics shape the influence of AI awareness (11, 13). This study addresses this gap by conceptualizing trait resilience as a pivotal psychological buffer, enabling the identification of employees with varying susceptibility to the negative implications of AI awareness.

This study’s fourth contribution extends research on work-to-family conflict by introducing technology-related stressors as a novel antecedent, with a particular emphasis on AI awareness. Prior research has predominantly explored WFC drivers in terms of personal characteristics, such as personality traits and resilience (39, 40), psychological capital (41, 42), as well as organizational factors, such as job demands, variable work hours (43), and leadership support (14, 44). However, there has been limited focus on stressors emerging from technological advancements (6), which are becoming increasingly significant in today’s workplaces. This study uncovers a previously unrecognized pathway through which technology-driven stressors, such as AI integration, intensify work–family conflict, thereby underscoring the urgency for organizations to develop specialized interventions that bolster employees’ capacity to adapt. By situating AI awareness within the broader spectrum of modern technological challenges, these insights reframe the understanding of work–life interplay and lay the groundwork for future empirical inquiries into strategies that can buffer the adverse consequences of emerging workplace technologies.

### 5.2 Practical implication

This study presents valuable practical insights. As AI technologies become increasingly integrated into service organizations, it is essential for managers to address not only consumer perceptions but also employees’ perspectives and attitudes toward AI. Our research demonstrates that AI awareness extends its influence beyond the workplace, significantly exacerbating work-to-family conflict among employees. Consequently, it becomes crucial for organizations adopting or expanding their use of artificial intelligence to take preemptive measures. To effectively buffer the negative impact of AI awareness on the work–family interface, a two-pronged approach is essential: fostering a genuinely supportive work environment and actively seeking to understand employee perceptions of these new technologies. Such an approach enables leadership to neutralize unintended adverse outcomes while simultaneously maximizing the strategic benefits of AI integration.

Second, organizations should take proactive measures to counteract the mental pressures triggered by employees’ awareness of AI technologies. Recent empirical evidence highlights the cascading risks of AI integration; for instance, a study by Zheng and Zhang (45) insightfully demonstrated that AI awareness can trigger a serial mechanism involving job insecurity and work interference with family, ultimately leading to emotional exhaustion. Drawing on these findings, it is critical for leadership teams to disrupt this negative chain by designing tailored support systems, training programs, and wellness initiatives aimed at sustaining psychological resilience. Systematic and periodic assessments of workforce well-being will allow early detection of potential issues and the implementation of preventive actions. Specifically, as suggested by Zheng and Zhang (45), transparent communication regarding AI’s role and the provision of retraining programs can effectively alleviate the job insecurity that often precipitates family interference. Organizations can further help employees maintain a healthier work–family balance by promoting flexible work arrangements and encouraging periods of digital disconnection from AI-driven tasks. Encouraging employees to engage in restorative leisure activities post-work can further enhance psychological detachment and recovery.

Finally, given the increasing integration of AI in frontline service roles, hospitality organizations should consider resilience as a key criterion when selecting employees for positions that involve frequent AI interactions. Specifically, during the recruitment process, firms can incorporate resilience assessments to identify candidates who are better equipped to manage AI-related stress and maintain psychological detachment from work. Additionally, for existing employees, targeted resilience-building initiatives, such as stress management training, adaptive coping workshops, and cognitive reframing programs, can help strengthen their ability to navigate AI-driven workplace transformations. Furthermore, mentorship programs that pair employees with experienced colleagues who have successfully adapted to AI-related changes can provide practical guidance and emotional support. By adopting these strategies, organizations can foster a more resilient workforce, ensuring that frontline employees are better prepared to handle AI awareness stressors while maintaining work–family balance.

### 5.3 Limitations

The interpretation of this study’s findings must acknowledge two principal boundaries. The first is methodological, stemming from our use of self-reported measures, which leaves the data susceptible to the influence of common method variance. The second boundary relates to generalizability; our sample was drawn exclusively from China’s hospitality sector, a unique environment shaped by collectivist norms. The specificity of this organizational and cultural context means that caution is warranted when extrapolating the results. To move beyond these limitations, future research should endeavor to test the proposed relationships within a broader spectrum of industries and cultures, thereby providing a more definitive assessment of the current findings’ robustness and applicability.

Third, although this study offers important insights into the buffering role of trait resilience in alleviating AI-induced stress, it does not address other potential individual characteristics that might influence employees’ responses to AI awareness. Future research could incorporate a temporal distance perspective to explore how variations in self-distancing tendencies affect employees’ capacity to detach psychologically from AI-related stressors. From the standpoint of self-distancing theory (46), individuals who adopt a self-distanced rather than a self-immersed orientation are generally more capable of disengaging from stressors and regulating their emotional reactions. Traits linked to temporal distance—such as future-oriented thinking or the ability to adopt a broader, long-term perspective—may determine the extent to which employees can relieve the pressures arising from AI awareness. Examining such differences would yield a more refined understanding of how employees cognitively manage AI-induced stress while sustaining work–family balance.

## Data Availability

The raw data supporting the conclusions of this article will be made available by the authors, without undue reservation.

## References

[1] Pan S-Y Lin Y Wong JWC. The dark side of robot usage for hotel employees: an uncertainty management perspective. Tour Manag. (2025) 106:104994. doi: 10.1016/j.tourman.2024.104994

[2] Teng R Zhou S Zheng W Ma C. Artificial intelligence (AI) awareness and work withdrawal: evaluating chained mediation through negative work-related rumination and emotional exhaustion. Int J Contemp Hosp Manag. (2023) 36:2311–26. doi: 10.1108/IJCHM-02-2023-0240, 35579975

[3] Frey CB Osborne MA. The future of employment: how susceptible are jobs to computerisation? Technol Forecast Soc Change. (2017) 114:254–80. doi: 10.1016/j.techfore.2016.08.019

[4] Anthony C Bechky BA Fayard A-L. “Collaborating” with AI: taking a system view to explore the future of work. Organ Sci. (2023) 34:1672–94. doi: 10.1287/orsc.2022.1651, 19642375

[5] He C Teng R Song J. Linking employees’ challenge-hindrance appraisals toward AI to service performance: the influences of job crafting, job insecurity and AI knowledge. Int J Contemp Hosp Manag. (2023) 36:975–94. doi: 10.1108/IJCHM-07-2022-0848, 35579975

[6] Zhou S Yi N Rasiah R Zhao H Mo Z. An empirical study on the dark side of service employees’ AI awareness: behavioral responses, emotional mechanisms, and mitigating factors. J Retail Consum Serv. (2024) 79:103869. doi: 10.1016/j.jretconser.2024.103869

[7] Brougham D Haar J. Smart technology, artificial intelligence, robotics, and algorithms (STARA): employees’ perceptions of our future workplace. J Manage Organ. (2018) 24:239–57. doi: 10.1017/jmo.2016.55

[8] Teng H-Y Li M-W Chen C-Y. Does smart technology, artificial intelligence, robotics, and algorithm (STARA) awareness have a double-edged-sword influence on proactive customer service performance? Effects of work engagement and employee resilience. J Hosp Market Manag. (2025) 34:443–66. doi: 10.1080/19368623.2025.2449853, 41307611

[9] Cheng M Zhang L Wang H. The effect of artificial intelligence awareness on frontline service employees’ silence: the roles of psychological contract breach and moral identity. Int J Contemp Hosp Manag. (2025) 37:1845–61. doi: 10.1108/IJCHM-07-2024-0968, 35579975

[10] Mo Z Liu MT Ma Y. How AI awareness can prompt service performance adaptivity and technologically-environmental mastery. Tour Manag. (2024) 105:104971. doi: 10.1016/j.tourman.2024.104971

[11] He C Xiong H Cai W Song J. How does AI awareness affect employees’ voice behavior in the service industry? A transactional theory of stress perspective. Int J Contemp Hosp Manag. (2025) 37:1662–80. doi: 10.1108/IJCHM-04-2024-0618, 35579975

[12] Lin Q He L. Does artificial intelligence (AI) awareness affect employees in giving a voice to their organization? A cross-level model. Int J Hosp Manag. (2024) 123:103947. doi: 10.1016/j.ijhm.2024.103947

[13] Wu T-J Liang Y Wang Y. The buffering role of workplace mindfulness: how job insecurity of human-artificial intelligence collaboration impacts employees’ work–life-related outcomes. J Bus Psychol. (2024) 39:1395–411. doi: 10.1007/s10869-024-09963-6

[14] Ye Y Wu L-Z Lyu Y Liu X. How to make the work-family balance a reality among frontline hotel employees? The effect of family supportive supervisor behaviors. Int J Hosp Manage. (2024) 120:103746. doi: 10.1016/j.ijhm.2024.103746

[15] Grzywacz JG Marks NF. Reconceptualizing the work–family interface: an ecological perspective on the correlates of positive and negative spillover between work and family. J Occup Health Psychol. (2000) 5:111–26. doi: 10.1037/1076-8998.5.1.111, 10658890

[16] Kuriakose V Sreejesh S. Co-worker and customer incivility on employee well-being: roles of helplessness, social support at work and psychological detachment- a study among frontline hotel employees. J Hosp Tour Manag. (2023) 56:443–53. doi: 10.1016/j.jhtm.2023.07.009

[17] Yang F Lu M Huang X. Customer mistreatment and employee well-being: a daily diary study of recovery mechanisms for frontline restaurant employees in a hotel. Int J Hosp Manage. (2020) 91:102665. doi: 10.1016/j.ijhm.2020.102665

[18] Schulz AD Schöllgen I Fay D. The role of resources in the stressor–detachment model. Int J Stress Manag. (2019) 26:306. doi: 10.1037/str0000100

[19] Sonnentag S Fritz C. Recovery from job stress: the stressor-detachment model as an integrative framework. J Organ Behav. (2015) 36:S72–S103. doi: 10.1002/job.1924

[20] Parker SL Jimmieson NL Walsh AJ Loakes JL. Trait resilience fosters adaptive coping when control opportunities are high: implications for the motivating potential of active work. J Bus Psychol. (2015) 30:583–604. doi: 10.1007/s10869-014-9383-4

[21] Demsky CA Ellis AM Fritz C. Shrugging it off: does psychological detachment from work mediate the relationship between workplace aggression and work-family conflict? J Occup Health Psychol. (2014) 19:195–205. doi: 10.1037/a0035448, 24635738

[22] Bakir S Dogru T Bilgihan A Ayoun B. AI awareness and employee-related outcomes: a systematic review of the hospitality literature and a framework for future research. Int J Hosp Manag. (2025) 124:103973. doi: 10.1016/j.ijhm.2024.103973

[23] Etzion D Eden D Lapidot Y. Relief from job stressors and burnout: reserve service as a respite. J Appl Psychol. (1998) 83:577–85. doi: 10.1037/0021-9010.83.4.577, 9729927

[24] Sonnentag S Fritz C. The recovery experience questionnaire: development and validation of a measure for assessing recuperation and unwinding from work. J Occup Health Psychol. (2007) 12:204–21. doi: 10.1037/1076-8998.12.3.204, 17638488

[25] Hobfoll SE Halbesleben J Neveu J-P Westman M. Conservation of resources in the organizational context: the reality of resources and their consequences. Annu Rev Organ Psychol Organ Behav. (2018) 5:103–28. doi: 10.1146/annurev-orgpsych-032117-104640

[26] Allen TD Johnson RC Saboe KN Cho E Dumani S Evans S. Dispositional variables and work–family conflict: a meta-analysis. J Vocat Behav. (2012) 80:17–26. doi: 10.1016/j.jvb.2011.04.004

[27] Hetrick AL Haynes NJ Clark MA Sanders KN. The theoretical and empirical utility of dimension-based work–family conflict: a meta-analysis. J Appl Psychol. (2024) 109:987–1003. doi: 10.1037/apl0000552, 37289526

[28] Adkins CL Premeaux SF. Spending time: the impact of hours worked on work–family conflict. J Vocat Behav. (2012) 80:380–9. doi: 10.1016/j.jvb.2011.09.003

[29] Zhao J Hu E Han M Jiang K Shan H. That honey, my arsenic: the influence of advanced technologies on service employees’ organizational deviance. J Retail Consum Serv. (2023) 75:103490. doi: 10.1016/j.jretconser.2023.103490

[30] Wang S Lin X Wu J. The effect of abusive supervision variability on work–family conflict: the role of psychological detachment and optimism [original research]. Front Psychol. (2023) 13:973634. doi: 10.3389/fpsyg.2022.973634 36733879 PMC9888312

[31] Raper MJ Brough P Biggs A. Is it all about the personal resources? The moderating role of resilience on daily stress appraisal and emotion. Work Stress. (2024) 38:248–69. doi: 10.1080/02678373.2023.2283217

[32] Hao S Hong W Xu H Zhou L Xie Z. Relationship between resilience, stress and burnout among civil servants in Beijing, China: mediating and moderating effect analysis. Pers Individ Differ. (2015) 83:65–71. doi: 10.1016/j.paid.2015.03.048

[33] Shi X Gordon S Tang C-H. Momentary well-being matters: daily fluctuations in hotel employees’ turnover intention. Tour Manag. (2021) 83:104212. doi: 10.1016/j.tourman.2020.104212

[34] Cho S Kim S. Does a healthy lifestyle matter? A daily diary study of unhealthy eating at home and behavioral outcomes at work. J Appl Psychol. (2022) 107:23–39. doi: 10.1037/apl0000890, 33764080

[35] Smith BW Dalen J Wiggins K Tooley E Christopher P Bernard J. The brief resilience scale: assessing the ability to bounce back. Int J Behav Med. (2008) 15:194–200. doi: 10.1080/10705500802222972, 18696313

[36] Spector PE Jex SM. Development of four self-report measures of job stressors and strain: interpersonal conflict at work scale, organizational constraints scale, quantitative workload inventory, and physical symptoms inventory. J Occup Health Psychol. (1998) 3:356–67. doi: 10.1037/1076-8998.3.4.356, 9805281

[37] Bono JE Foldes HJ Vinson G Muros JP. Workplace emotions: the role of supervision and leadership. J Appl Psychol. (2007) 92:1357–67. doi: 10.1037/0021-9010.92.5.1357, 17845090

[38] Fornell C Larcker DF. Evaluating structural equation models with unobservable variables and measurement error. J Mark Res. (1981) 18:39–50. doi: 10.1177/002224378101800104

[39] Dönmez FG Gürlek M Karatepe OM. Does work-family conflict mediate the effect of psychological resilience on tour guides’ happiness? Int J Contemp Hosp Manag. (2024) 36:2932–54. doi: 10.1108/IJCHM-01-2023-0077

[40] Li W-D Wang J Allen T Zhang X Yu K Zhang H . Getting under the skin? Influences of work–family experiences on personality trait adaptation and reciprocal relationships. J Pers Soc Psychol. (2024) 126:694–718. doi: 10.1037/pspp0000476, 37561453

[41] Lim WM Cabral C Malik N Gupta S. How does ethical climate enhance work–family enrichment? Insights from psychological attachment, psychological capital and job autonomy in the restaurant industry. Int J Contemp Hosp Manag. (2022) 35:1713–37. doi: 10.1108/IJCHM-03-2022-0383, 35579975

[42] Sarwar F Panatik SA Sukor MSM Rusbadrol N. A job demand–resource model of satisfaction with work–family balance among academic faculty: mediating roles of psychological capital, work-to-family conflict, and enrichment. SAGE Open. (2021) 11:21582440211006142. doi: 10.1177/21582440211006142

[43] Cho H Lambert SJ Ellis E Henly JR. How work hour variability matters for work-to-family conflict. Work Employ Soc. (2024) 38:1611–35. doi: 10.1177/09500170231218191

[44] Hunsaker WD. Spiritual leadership and work–family conflict: mediating effects of employee well-being. Personnel Rev. (2021) 50:143–58. doi: 10.1108/PR-04-2019-0143

[45] Zheng J Zhang T. Association between AI awareness and emotional exhaustion: the serial mediation of job insecurity and work interference with family. Behav Sci. (2025) 15:401. doi: 10.3390/bs15040401, 40282023 PMC12024253

[46] Kross E Ong M Ayduk O. Self-reflection at work: why it matters and how to harness its potential and avoid its pitfalls. Annu Rev Organ Psychol Organ Behav. (2023) 10:441–64. doi: 10.1146/annurev-orgpsych-031921-024406

