# Supplementary material for: AI awareness and the breakdown of daily recovery: a spillover pathway to work–family strain
Source: Front Public Health. 2026 Jan 13;13:1738073. doi: 10.3389/fpubh.2025.1738073 (PMC12835287; doi:10.3389/fpubh.2025.1738073)
Supplement: Supplementary file 1 [file Table_1.docx]

Appendix

Appendix Table A1

| Day-level variables | Intercept (γ00) | Within-person variance (σ2) | Between-person variance (τ00) | Variability between-person (%) | Variability within-person (%) |
| --- | --- | --- | --- | --- | --- |
| AIA | 3.245*** | 0.818 | 0.343 | 29.54 | 70.46 |
| PD | 2.968*** | 0.461 | 0.189 | 29.08 | 70.92 |
| WFC | 2.963*** | 0.919 | 0.356 | 27.92 | 72.08 |
| JD | 3.022*** | 0.744 | 0.225 | 23.22 | 76.78 |
| NE | 2.796*** | 1.11 | 0.35 | 23.97 | 76.03 |

*Note:* AIA (AI Awareness), PD (Psychological Detachment), WFC (Work-Family Conflict), TR (Trait Resilience), JD (Job Demand), and NE (Negative Emotions).
